# First Metataxonomic Characterisation of Gut Microbiota of Swordfish ( *Xiphias gladius* )

**DOI:** 10.1111/1758-2229.70199

**Published:** 2025-11-05

**Authors:** Alessandro Truant, Federica Giacometti, Carmen Losasso, Arianna Peruzzo, Sara Petrin, Irene Zancato, Vincenzo Di Leva, Valerio Giaccone

**Affiliations:** ^1^ Department of Animal Medicine, Production and Health University of Padova Legnaro Padova Italy; ^2^ Istituto Zooprofilattico Sperimentale Delle Venezie Laboratory of Microbial Ecology and Genomics Legnaro Italy; ^3^ Fiorital SPA Venezia Italy

**Keywords:** gut microbiota, metataxonomic characterisation, next‐generation sequencing, swordfish ( *Xiphias gladius* )

## Abstract

Swordfish ( *Xiphias gladius* ) is a large, migratory apex predator with a carnivorous diet, occupying a top position in the marine food chain. Although it is a valuable teleost pelagic fish with a significant commercial value, its gut microbiota has never been studied. The gut microbiota of 100 individuals was characterised by sequencing the V3–V4 region of the bacterial 16S rRNA gene. Gut microbiota findings were classified with consideration to diversity, taking into account their weight (10–20; 21–30; over 31 kg) and the FAO fishing areas in which they were caught (FAO 27, 34, 37.1.1 areas). Significant differences in the alpha diversity were observed among the weight categories for all metrics examined (except for the evenness index) and only by Shannon's index among the FAO fishing areas. Beta‐diversity analysis revealed no significant differences. The phylum Pseudomonadota dominated the swordfish gut microbiota, followed by Fusobacteriota. *Photobacterium* was the most abundant genus across all weight categories and FAO fishing areas. Smaller fishes showed a less rich and diverse gut microbiota, dominated almost exclusively by *Photobacterium.* Conversely, *Pseudoalteromonas, Psychrobacter, Psychrilyobacter*, and *Cetobacterium* appeared to increase in abundance with fish weight. Although *Photobacterium* was dominant across the different FAO fishing areas, distinctive microbial community compositions were observed: *Cetobacterium* was more prevalent in FAO 27, while *Pseudoalteromonas* was more prevalent in the other areas. Unlike the gut microbiota of other marine fish species, *Vibrio* and *Lactobacillus* were largely absent. This study represents the first metataxonomic characterisation of the gut microbiota of swordfish using next‐generation sequencing.

## Graphical abstract

This is the first metataxonomic characterisation of gut microbiota of swordfish using next‐generation sequencing. Findings show significant differences in the alpha diversity among the weight categories and the FAO fishing areas. The phylum Pseudomonadota dominated the swordfish gut microbiota, followed by Fusobacteriota. Photobacterium resulted in the most abundant genus across all weight categories and FAO fishing areas. Smaller fishes showed a less rich and diverse gut microbiota, dominated almost exclusively by Photobacterium. Conversely, Pseudoalteromonas, Psychrobacter, Psychrilyobacter, and Cetobacterium appeared to increase in abundance with fish weight. Although Photobacterium was dominant also across the different FAO fishing areas, distinctive microbial community compositions were observed, with Cetobacterium more prevalent in FAO 27 and Pseudoalteromonas in the other areas.

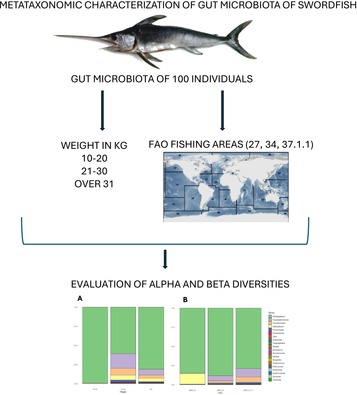

## 1 Introduction

Swordfish ( *Xiphias gladius* ) is a large, silver teleost pelagic fish with a distinctive long, flat rostrum on its upper jaw, forming the typical flat and sharp ‘sword’, approximately one‐third of its body length. It is found in tropical, temperate, and sometimes cold waters of all oceans, including the Mediterranean and its adjacent waters (EUMOFA 2019). The swordfish is a valuable fish species, both ecologically, as it is considered a good candidate for monitoring ecosystem changes due to its generalist and opportunistic feeding behaviour (Fernández‐Corredor et al. 2023), and commercially. Indeed, swordfish is commercially important to several European Union (EU) fleets fishing in the Atlantic Ocean and the Mediterranean Sea. With global catches relatively stable at 100–120 kilotonnes in recent years (https://www.fao.org/fishery/en/aqspecies/2503/en), EU fleets account for nearly one‐third of the global supply. Spain is by far the largest producer and, to a lesser extent, Italy, with over 15,000 and 2000 t landed in 2022, respectively (EUMOFA 2024). However, in the EU, the main market for swordfish is Italy, being among the top 10 species consumed at the household level (EUMOFA 2023) and showing a nominal value of million euros in 2022 (EUMOFA 2024). Its consumption has been growing in an unsustainable way in recent decades, and the consequent intensive fishing worldwide has compromised swordfish marine stocks. Both local and international policies have been starting to protect this species in recent years (ICCAT 2016). Swordfish populations are subdivided mainly on an ocean‐basin scale, with a unique Mediterranean stock distinct from the Atlantic ones, exhibiting different growth and maturity characteristics, and in which two other sub‐stocks can be observed, respectively, North and South Atlantic (Kotoulas et al. 1995). The swordfish is a large, migratory apex predator with a carnivorous diet and occupies a top position in the marine food chain, being an extremely versatile predator capable of exploiting various trophic resources. Indeed, it feeds on a broad spectrum of prey, comprising mainly teleost fish, primarily pelagic fishes at the juvenile stage, and later, as adults, predates on crustaceans and cephalopods (Abbate et al. 2017), depending on fish size, area, and season, and also invertebrates (Navarro et al. 2017). This species is renowned for achieving substantial dimensions, with adult individuals attaining lengths of up to 3 to 4 m and weighing over 400 kg, and for crossing long distances in a short time, appearing to prefer different habitats for breeding and feeding (Canese et al. 2008).

It is well known that the adaptation of vertebrates to their environment is strictly related to their capacity for feeding and that fish have a unique and intimate interaction with their surrounding environment and, in turn, with the microorganisms that co‐exist there. The alimentary canal of fish varies greatly with differing life histories, ecology, and environmental factors related to fish biology. Filter feeders, parasites, and predators, as well as herbivorous and carnivorous fish, exist, and each has an appropriately adapted and different digestive system, including digestive organs that may or may not be present (Egerton et al. 2018). Teleosts' gut, as well as skin mucus and gills, supports abundant populations of bacteria that impact the overall health and physiology of the host. Fish intestines, in particular, harbour large and diverse populations of bacteria (Givens et al. 2015), which are the dominant microbiota of fish intestines (Rombout et al. 2011). Literature on fish gut microbiota has attempted to provide an understanding of many areas, and mainly of the diverse fish species, given that there are approximately 33,700 fish species (only teleosts are more than 28,000 species) on Earth, accounting for over half of the total number of vertebrate species. The term ‘microbiota’ comprises the entire collection of microorganisms in a specific niche, such as the fish gut, and a 16S rRNA survey resulted in the analytical approach used to taxonomically identify these microorganisms in a specific environment (Berg et al. 2020). However, these studies varied considerably in terms of the species investigated, methodologies, and sample collection (e.g., fore‐, mid‐, and high gut), as well as analytical methods (culture‐dependent versus culture‐independent methods), with consequent difficulties in comparing results and extrapolating the true level of diversity. These conflicting findings were undoubtedly a feature of the diversity that exists among fish, but there was also variability among individuals of the same species. Factors influencing the intra‐and inter‐species diversity found in gut microbiota studies include life stage, diet, season, habitat, captive state, sex, and phylogeny (Wang et al. 2018), even if trophic level, habitat, and possibly host phylogeny are the most likely influencers (Sullam et al. 2012). The influence of diet on gut microbiota is a logical link, as well as that with the surrounding water, reflecting diverse environmental compositions (Dehler et al. 2017). However, microbiota variability among individuals, across a great number of hosts, is still considered not to have been systematically investigated (Reese and Dunn 2018).

Worldwide, there is a large body of research on the biology (diet, growth, and reproduction), migrations, genetic structure of fish populations, and heavy metal accumulation of swordfish, but only a few papers address the microbiological safety of their meat (Indio et al. 2024; Zakrzewski et al. 2023; Chang et al. 2008; Boutin et al. 1998) and, to our knowledge, to date, no study has yielded a 16S rRNA next‐generation sequencing analysis in the gut of this fish species. Herein, this study addresses a metataxonomic analysis of swordfish gut and explores the characterisation of the intestinal microbiota of wild individuals in relation to the fish size and the different fishing areas in which they were caught.

## 2 Materials and Methods

### 2.1 Fish Samplings and Gut Content Collection

A total of 100 swordfish individuals were included in this study. Fish sampling was conducted at a fish processing facility in Venice, Italy, specialising in the marketing (over 40,000 tonnes of fish annually as wholesalers of fresh, frozen, and blast‐chilled fish) as well as the processing and packaging of fish products. Data pertaining to product traceability and labelling EC No 178/2002; Regulation EC No 1379/2013 were used to collate the commercial designation and scientific name of the fish species and the area where the fish were caught. The investigated swordfish individuals weighed between 10 and 37 kg and were caught in three different FAO fishing areas: FAO 27 (Atlantic, Northeast), FAO 34 (Atlantic, Eastern Central), and, within FAO 37 (Mediterranean and Black Sea) and the Western Mediterranean subarea (Subarea 37.1), the Balearic division (Division 37.1.1). The swordfish individuals were divided into groups based on fish weight (10–20 , 21–30 , and > 31 kg) and FAO fishing areas (FAO 27, 34, and 37.1.1). Table 1 summarises the classification of the swordfish individuals.

**TABLE 1:** Details of the overall swordfish individuals involved into the study and classified into different groups by the fish weight and the FAO fishing areas.

FAO fishing area	Fish weight (kgs)			
	10–20	21–30	> 30	Sum
FAO 27	1	10	14	25
FAO 34	25	7	8	40
FAO 37.1.1	20	8	7	35
Sum	46	25	29	100

The fishes were processed in a timely manner in order to reduce the change in composition of gut microbial communities. Before dissection, according to literature, 70% ethanol was applied to the body surface of the fish samples to decontaminate the skin and scales, not the incision or internal tissues (Xia et al. 2014; Huang et al. 2020; Le and Wang 2020; Song et al. 2023). From each swordfish individual, the digestive tract from the stomach to the hindgut was removed intact. Swordfish was kept in a vertical position on the slaughter line; an abdominal incision was performed using a sterile knife to remove offal and the whole visceral package. For each swordfish individual, the knife was replaced to ensure the sterility of the procedure. The gut was directly transferred, by gravitational fall, into a sterile bag handled by the laboratory technician, avoiding any contact with non‐sterile surfaces and ensuring the traceability of each individual sample. The entire process took place in a refrigerated environment at 4°C within the investigated fish processing facility. From each swordfish gut, two separate biological replicates of the faecal content were collected using swabs (FecalSwab, Copan Diagnostics Inc., Brescia, Italy), transported under chilled conditions (at 4°C) to the Laboratory of Microbial Ecology at the Istituto Zooprofilattico Sperimentale delle Venezie and processed within 36 h.

### 2.2 DNA Extraction and 16S rRNA Sequencing

DNA from the gut content samples was extracted starting from 200 μL of faecal content with a column‐based kit, QIAamp DNA Mini Kit (QIAGEN, Hilden, Germany), following the manufacturer's instructions. Thermal lysis was carried out for 2 h, and RNaseA (100 mg/mL) was added to each sample to ensure RNA‐free preparation. Total DNA was resuspended in 200 μL of nuclease‐free water and stored at−20°C. The hypervariable V3 and V4 regions of the bacterial 16S rRNA gene of the qualified DNA samples were amplified by the primers Bact341F and Bact785R (Fwd: CCTACGGGNGGCWGCAG and Rev.: GACTACHVGGGTATCTAATCC) previously described by Klindworth and co‐authors (2013) and according to the Illumina 16S Metagenomic Sequencing Library Preparation protocol. The thermal cycling profile was as follows: 95°C for 3 min; 25 cycles of 95°C for 30 s, 56°C for 30 s, 72°C for 30 s; 72°C for 5 min; hold at 4°C. PCR clean‐up was performed by means of the Agencourt AMPure XP beads (Beckman Coulter Genomics, Indianapolis, IN, USA). Libraries for sequencing were prepared using the Nextera XT DNA Library Prep Kit (Illumina). The concentration of the libraries was determined with the Qubit fluorometric assay, and their quality was assessed with the Agilent 2200 TapeStation. Samples were pooled equimolarly, and the resulting library was sequenced on the Illumina MiSeq platform using a MiSeq 600 V3 cartridge (600 cycles, 2 × 300 bp, paired‐end reads).

### 2.3 Bioinformatic and Statistical Analysis

Raw sequence data were processed using the Quantitative Insights Into Microbial Ecology 2 (QIIME2) version 2020.2 pipeline. Reads were screened, trimmed, quality filtered, and denoised with DADA2 (Callahan et al. 2016) using the following parameters: p‐trunc‐len‐*f* = 284 and p‐trunc‐len‐*r* = 276. Amplicon Sequence Variants (ASVs) were converted into OTUs to reduce computational complexity of the data while still ensuring a sufficient level of detail. Operational taxonomic units (OTUs) were defined as those sequences with a similarity ≥ 97% among them. Each OTU was taxonomically assigned based on the SILVA database (Quast et al. 2012), and the obtained OTU‐table was then normalised following the Geometric Mean of Pairwise Ratio (GMPR) method (Chen et al. 2018). Statistical analyses were conducted using the *phyloseq* package (McMurdie and Holmes 2013). Alpha diversity analyses were performed on the pre‐processed count table and measured by means of the observed OTU metric, Pielou index, and Shannon index to describe the community structure. Beta diversity was evaluated with the Bray‐Curtis distance metric and visualised using the Non‐metric Multi‐Dimensional Scaling (NMDS) plot. Kruskal‐Wallis non‐parametric test was used to compare alpha diversity between different swordfish weight categories and between different FAO fishing areas. If significant, the pairwise comparison Wilcoxon test was performed by adjusting the *p*‐value for multiple comparisons using the false discovery rate (FDR) method (Benjamini and Hochberg 1995). PERMANOVA test was used to compare the beta‐diversity parameters between groups. Finally, the differential abundance analysis was performed using the ANCOM‐BC test at the genus level (Mandal et al. 2015). Graphical outputs were obtained by means of the *ggplot2* package (Villanueva and Chen 2019). R version 4.3.2 software (R Core Team 2023) was used to perform the above statistical analysis. *p*‐value < 0.05 is considered significant.

## 3 Results

The 16S rRNA metataxonomic sequencing of swordfish intestinal microbiota produced an output of a total of 21,395,047 reads, yielding an average of 211,227 reads per sample (standard deviation, SD = 48,008). After the quality trimming step, 3,297,390 sequences were retained for further analysis with a mean for the sample of 329,739 sequences (SD = 10,037) and then sorted into OTUs. The OTUs showed substantial variation across the samples, with counts ranging notably from 20 OTUs to 190,202 (in sample 65), on average.

Based on the phylogenetic classification using the SILVA database, the microbiota taxonomic composition at the phyla level was Pseudomonadota (92%) and Fusobacteriota (7%), and a small percentage (< 1%) was represented by other phyla such as Bacillota, Cyanobacteriota, and Mycoplasmatota. In relation to the microbiota taxonomic composition at the genus level, the most abundant genera within the phylum Pseudomonadota were represented by *Photobacterium* (91%), *Pseudoalteromonas* (7%), *Psychrobacter* (1.1%), *Vibrio* (0.3%), *Pseudomonas* (0.3%), and *Shewanella* (0.2%). The most abundant genera within the phylum Fusobacteriota were represented by *Psychrilyobacter* (52%) and *Cetobacterium* (46%), whereas within the phyla Bacillota and Mycoplasmatota, respectively, the genera *Tyzzerella* (0.62%) and *Ureaplasma* (0.2%) were represented.

**FIGURE 1:**
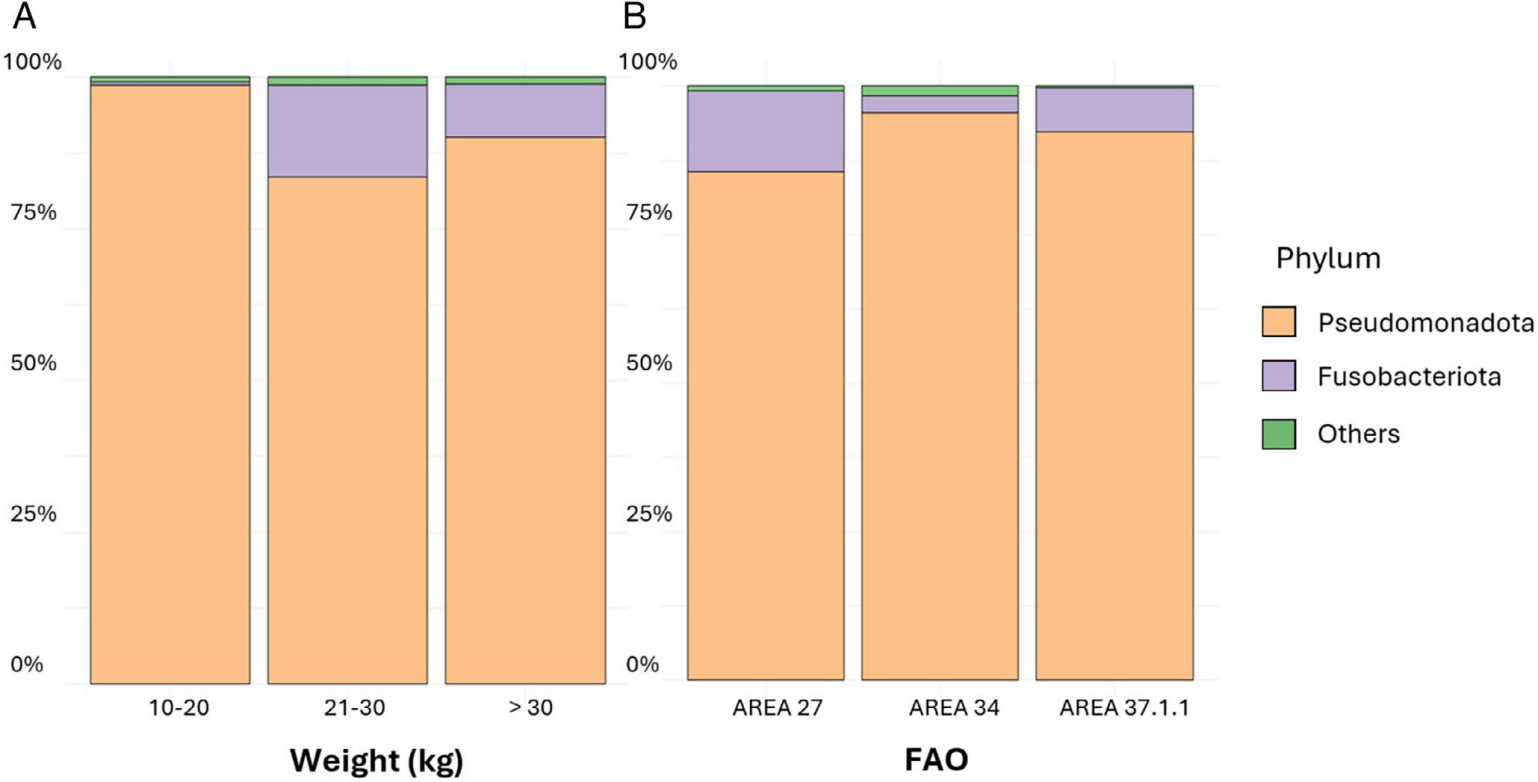
Barplots showing the relative abundance of the most abundant phyla identified in gut Swordfish splitted into the different categories, respectively (A) weight (10–20, 21–30, and over 30 kg) and (B) FAO fishing areas (FAO 27 Atlantic, Northeast, FAO 34 Atlantic, Eastern Central and FAO 37.1.1 Mediterranean and Black Sea, Balearic division).

The gut microbiota findings showed a variability considering the two investigated categories, namely, weight and FAO fishing area. Figure 1 shows the phyla identified in the gut of swordfish in terms of relative abundance. Pseudomonadota dominated the gut microbiota across all the weight categories (min 84%, max 99%, mean 91%) and FAO fishing areas (min 86%, max 95%, mean 91%). The differential abundance test revealed that, considering the different weights of the analysed fish, 10 taxa exhibit a statistically significantly higher abundance in fish weighing between 10 and 20 kg, 14 taxa in those weighing between 21 and 30 kg, and 11 taxa in fish weighing over 30 kg. Regarding the different FAO sampling areas, 11 taxa are statistically abundant in fish from FAO area 27, 15 in fish from FAO area 34, and 10 in those from FAO area 37.1.1.

**FIGURE 2:**
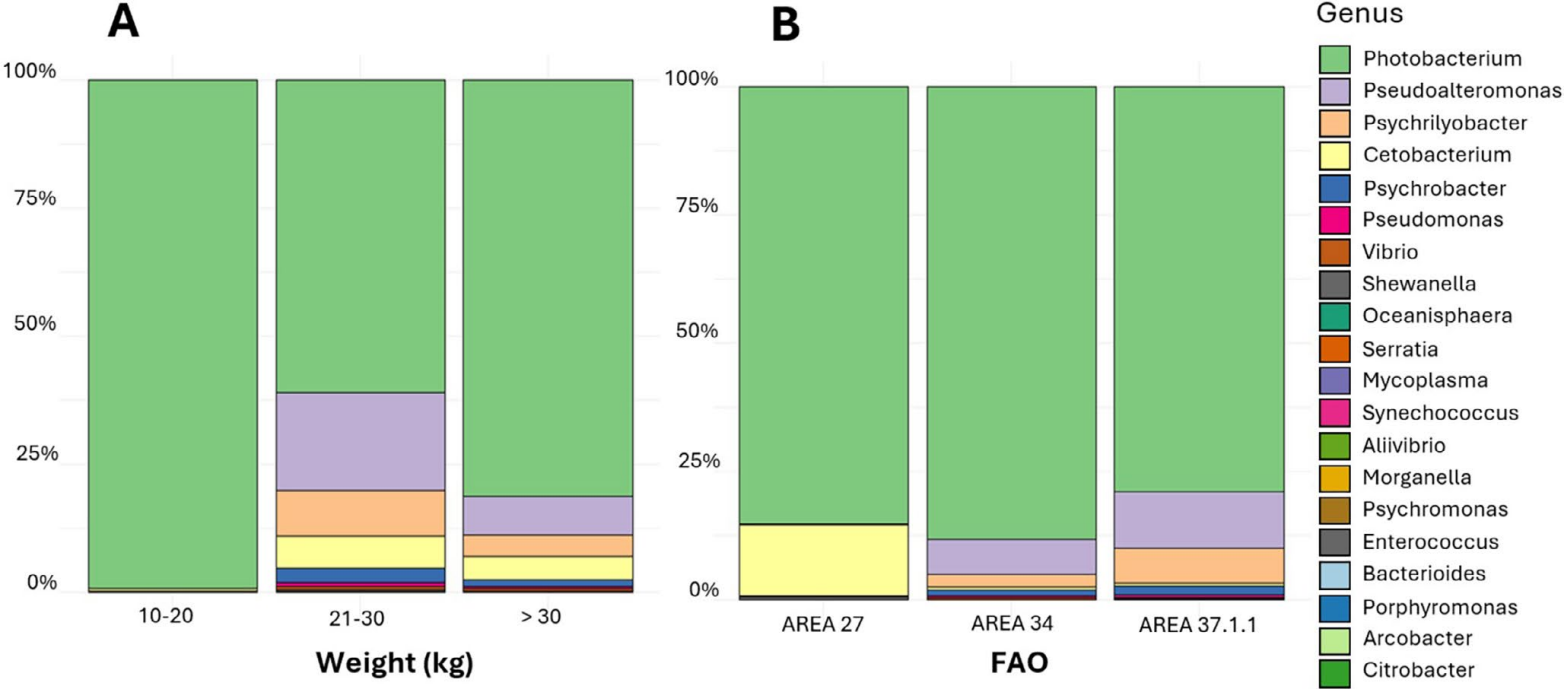
Barplots showing the relative abundance of the most abundant genera identified in gut Swordfish splitted into the different categories, respectively (A) weight (10–20, 21–30, and over 30 kg) and (B) FAO fishing areas (FAO 27 Atlantic, Northeast, FAO 34 Atlantic, Eastern Central and FAO 37.1.1 Mediterranean and Black Sea, Balearic division).

Figure 2 shows the most abundant identified genera in gut swordfish in terms of relative abundance and their variability within the investigated categories, weight and FAO fishing area. The genus *Photobacterium* dominated the gut microbiota across all the weight categories, reaching a maximum of 98.5% in smaller fishes (versus 60.2% and 80% in medium and larger fishes) and FAO fishing areas (min 78.6%, max 86.9%). However, among the Pseudomonadota phylum, all the other genera, namely *Pseudoalteromonas, Psychrobacter*, *Vibrio*, and *Pseudomonas*, show a lower relative abundance (< 0.1%) only in smaller fishes and in the FAO 27 area. Among Fusobacteriota, the presence of *Psychrilyobacter* seems to increase with the weight of the fish (< 0.1% in smaller fishes versus 8.9% and 4.2% in medium and larger fishes) as well as to be more abundant in FAO 34 and 37.1.1 areas (< 0.1% in FAO 27 versus 2.4% and 6.7% in FAO 34 and 37.1.1 areas). Also, the presence of *Cetobacterium* seems to increase with the weight of the fish (< 1% in smaller fishes versus 6.2% and 4.6% in medium and larger fishes), whereas, on the contrary, it is more abundant in FAO 27 area (13.7% in FAO 27 versus 0.5% and 0.65% in FAO 34 and 37.1.1 areas).

**FIGURE 3:**
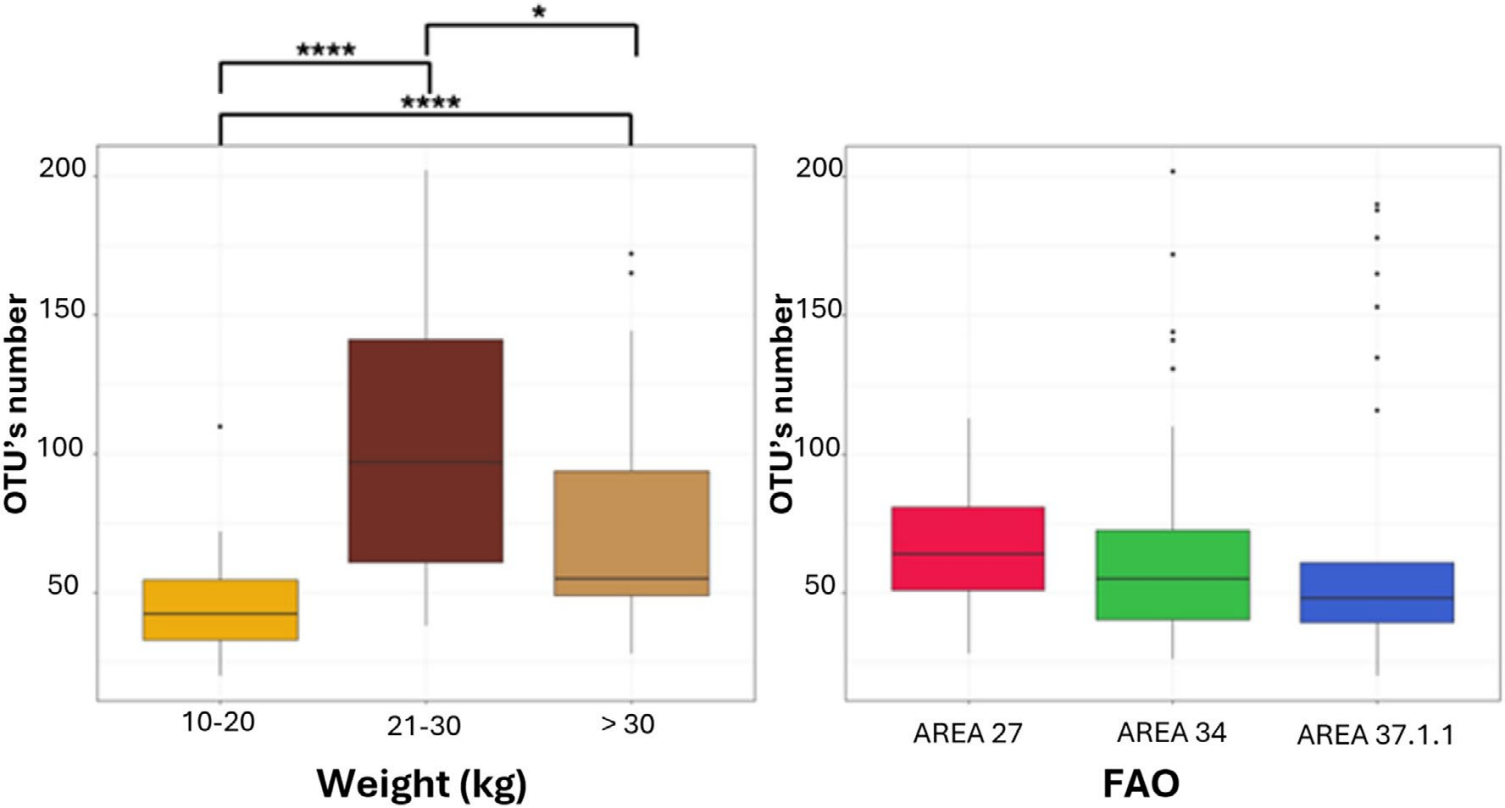
Boxplots of the number of identified OTUs in the overall gut samples divided by different weight (A) and different FAO area (B). Outliers are indicated by dots in each boxplot. The significance of differences between the groups is indicated by asterisks: *p < 0.05; **p < 0.01; ***p < 0.001; *p* < 0.0001.

**FIGURE 4:**
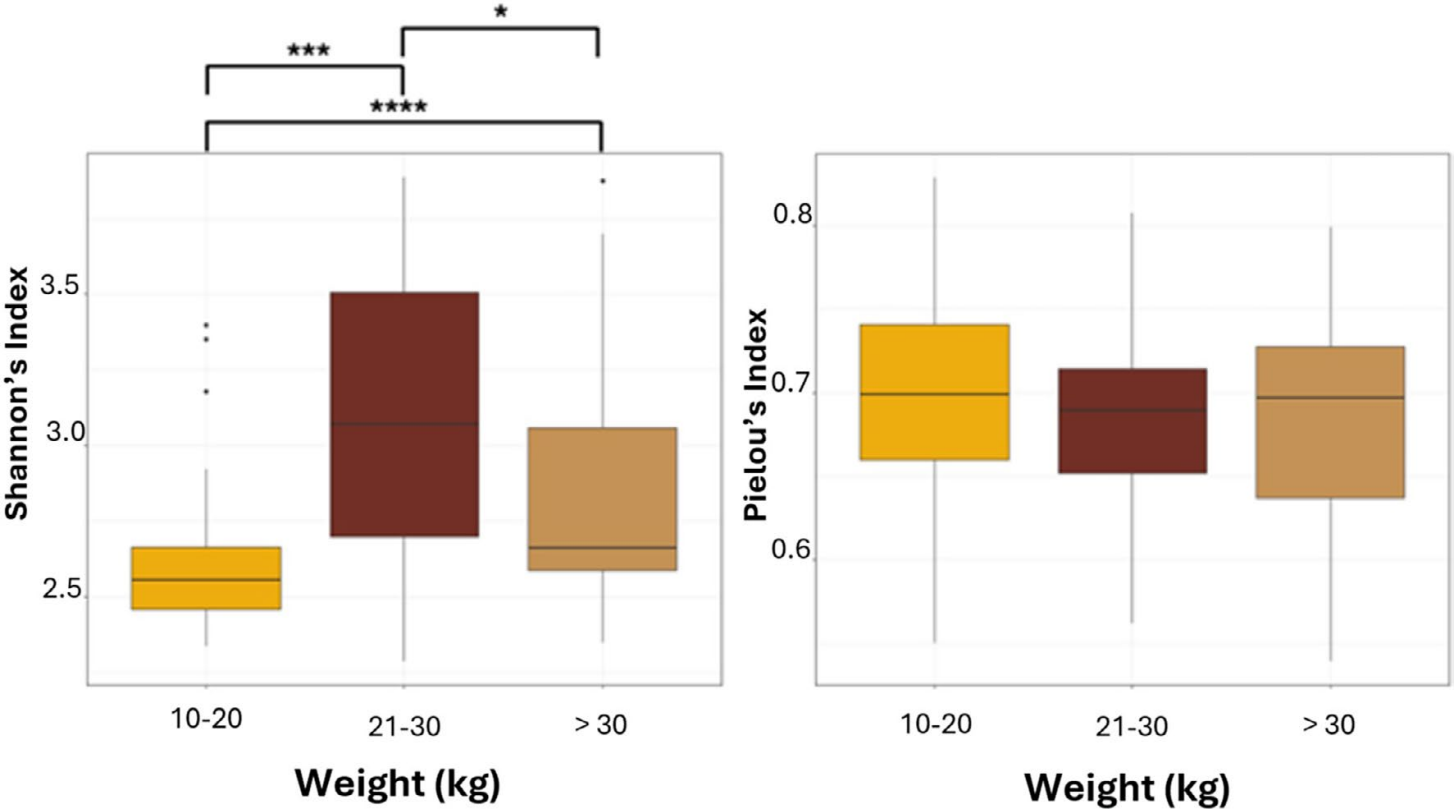
Microbiota diversity in different weight fish categories using Shannon and Pielou diversities indices; the colours of the boxplots represent the different swordfishes' weight categories. Outliers are indicated by dots in each boxplot. The significance of differences between the groups is indicated by asterisks: *p < 0.05; **p < 0.01; ***p < 0.001; *p* < 0.0001.

**FIGURE 5:**
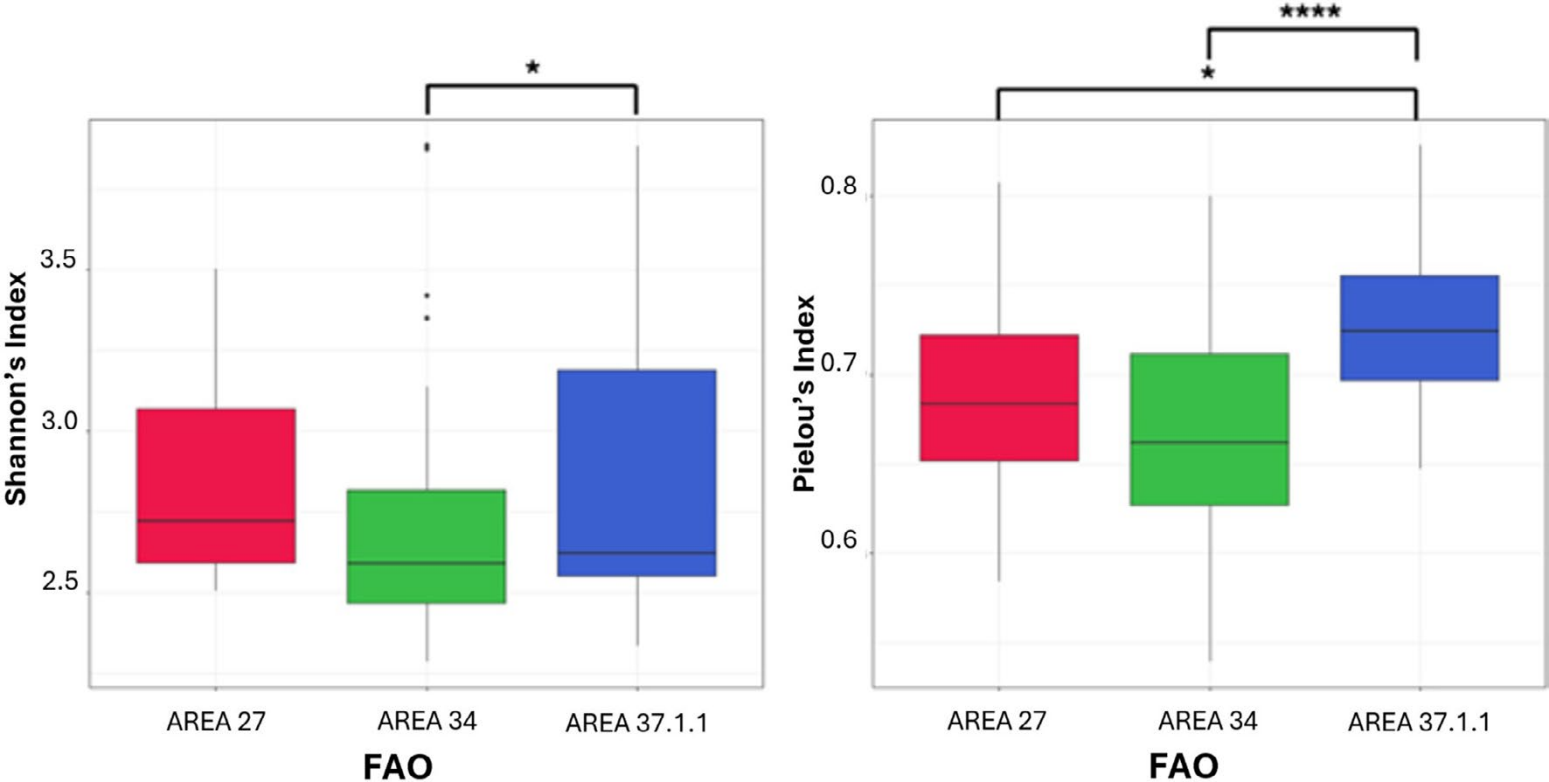
Microbiota diversity in different weight FAO fishing areas using Shannon and Pielou diversities indices; the colours of the boxplots represent the different FAO areas. Outliers are indicated by dots in each boxplot. The significance of differences between the groups is indicated by asterisks: *p < 0.05; **p < 0.01; ***p < 0.001; *p* < 0.0001.

In addition, alpha diversity metrics unveiled differences among the investigated categories among the overall 100 swordfish guts, namely, weight and FAO fishing area. In more detail, among the weight categories, statistical tests showed: (i) statistically significant lower OTUs count in smaller fishes compared to medium‐weight and larger fishes (respectively *p*‐value = 2.3e‐7 for 10–20 vs 21–30 and 7.1e‐5 for 10–20 vs. 31 + kg) (see Figure 3A); (ii) statistically significant lower biodiversity, in terms of Shannon index, in smaller fishes compared to medium and large fishes, with the most significant distinction between small and medium fish groups (10–20vs. 21–30 *p*‐value = 3.1e‐6; 10‐20vs. over 30 *p*‐value = 0.0017 and 21–30 vs. over 30 *p*‐value = 0.04); (iii) no statistical differences by the evenness index, revealing a uniform microbial taxa distribution across the three weight categories (*p* = 0.5987) (see Figure 4). Among the FAO fishing areas, statistical tests showed: (i) no significant difference between the identified OTUs across the samples (*p*‐value = 0.1402) (see Figure 3B); (ii) only the comparison between fishes from FAO area 34 and FAO area 37.1.1 revealed a significant lower Shannon's index in FAO area 34 than area 37.1.1 (*p*‐value = 0.034); (iii) statistically significant differences by the Pielou's index exist between fishes from FAO area 27 and FAO area 37.1.1 (lower in area 27) and between fishes from FAO area 34 and FAO area 37.1.1 (lower in FAO area 34) (respectively *p*‐value = 0.03 and *p*‐value = 8.8e‐5) (see Figure 5).

**FIGURE 6:**
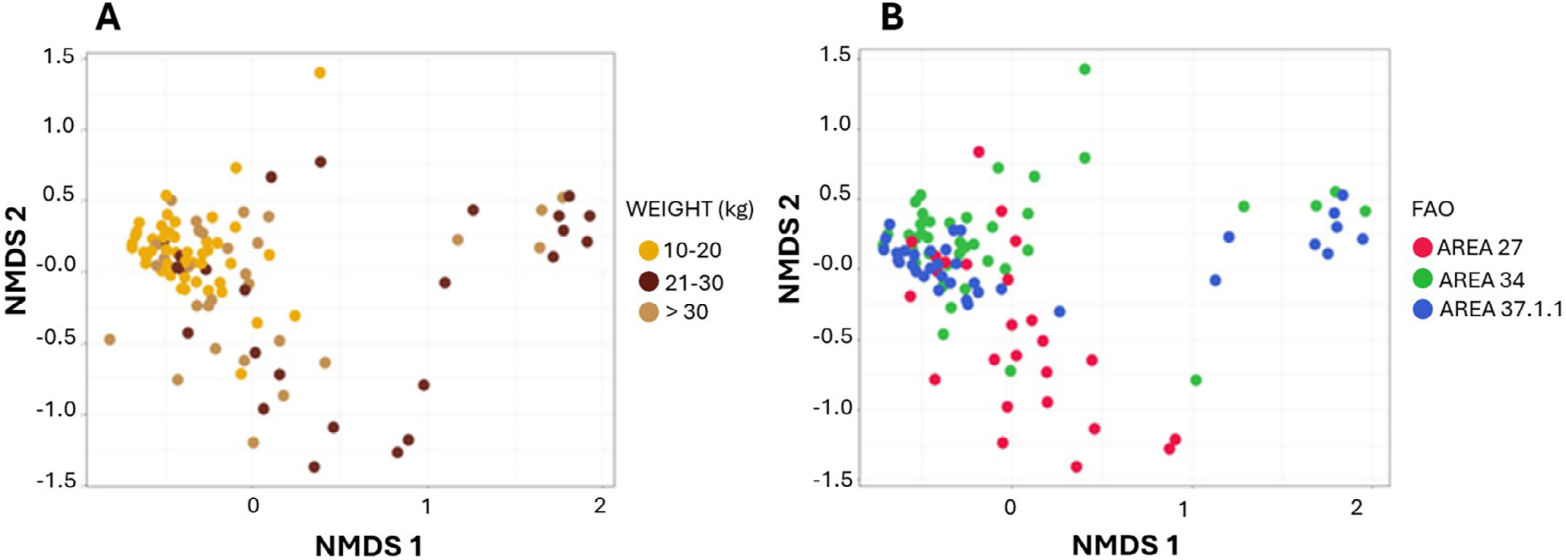
NMDS plots showing the weight category (A) and FAO fishing areas (B).

The beta‐diversity analysis revealed no significant differences in the taxonomic structure of the microbial community among the three weight categories and FAO fishing areas under consideration. Figure 6 shows the NMDS plot displays samples labelled by weight category and FAO fishing areas, with no discernible clusters observed. Nevertheless, the NMDS plot (Figure 6) shows that samples from small fish (yellow dots) tend to cluster more closely together compared to the other two weight categories, indicating a tighter grouping pattern despite the absence of statistically tested differences.

## 4 Discussion

While the literature has begun to elucidate the microbial characteristics of many fish species, available data on gut microbiota are still lacking for *Xiphias gladius* . This study identified distinctions in swordfish gut microbiota between individuals of different sizes and those caught in different FAO fishing areas, elucidating intra‐species microbiota diversity. Given that gut microbiota structure is primarily influenced by trophic level, habitat and host phylogeny (Sullam et al. 2012), it is of clear interest to understand the differences occurring in an apex predator and a highly migratory mesopelagic fish widely distributed in the Atlantic Ocean and Mediterranean Sea. Therefore, although swordfish are carnivorous fish, their habitat and diet cannot be considered uniform. The investigated swordfish were categorised into small, medium and large individuals based on the minimum conservation reference size and the average weight data reported for Mediterranean swordfish. Specifically, (Regulation (EU) 2019/1154 2019) on a multiannual recovery plan for Mediterranean swordfish, and namely sec. 2, Article 11 details the minimum conservation reference size for Mediterranean swordfish. It stipulates that, by way of derogation from Article 15(1) of Regulation (EU) No 1380/2013, it is prohibited to catch, retain on board, transship, land, transport, store, sell, or display or offer for sale Mediterranean swordfish, including in recreational fisheries, measuring less than 100 cm lower jaw to fork length or weighing less than 11.4 kg of live weight, or 10.2 kg of gilled and gutted weight. However, in the case of incidental catches below the minimum conservation reference size, exceptions are allowed only if such catches do not exceed 5% by weight or number of specimens of the total Mediterranean swordfish catch of the fishing vessels concerned. Furthermore, the weight categorisation reflects the swordfish size distribution recently observed in several areas of the Mediterranean Sea. Among 987 swordfish caught during the 2021 and 2022 fishing seasons, total round weights ranged from 5 to 128 kg, with a mean value of 33 kg (ICCAT 2016; Pappalardo and Pignalosa 2022). Based on 2019 catches, the size‐weight classes between 10 and 35 kg accounted for 63.40% of the total catches. Classes above 35 kg all contributed to the remaining 36.60% of the total catches, particularly for classes above 80 kg, where the yield was very low (ICCAT 2016; Pignalosa et al. 2019). Similarly, based on swordfish catches in the Sicily Region, Italy, between 1986 and 2010, the frequency distributions of the raw individual weight data were skewed but generally peaked in the size range 20–40 kg (min 20, max 79, geometric mean of 40 kg), with most of the individually caught swordfish aged 2–4 years (MacKenzie et al. 2022). Additionally, given the weight‐at‐age and maturity‐at‐age relationships (50% probability of maturity at age 3.5 years and 37 kg), the captured swordfish were a mix of larger juveniles and adults, with approximately half of the individuals being mature. However, the true probability of maturity‐at‐size or ‐age of swordfish in the Mediterranean is uncertain and likely differs throughout the region (MacKenzie et al. 2022). Therefore, the three investigated weight categories (small, medium, and large fish) reflect the real‐world scenarios in which swordfish are sold at fish markets, as well as the differentiation between juveniles (small fish) and adults (medium and large fish). Also, the three investigated FAO fishing areas have some peculiarities that differently characterised the swordfish habitats. Mediterranean swordfish have different biological characteristics compared to the Atlantic stock, with different growth parameters and sexual maturity reached at younger ages than in the Atlantic.

Overall, consistent with previous studies performed in the gut of marine and non‐herbivorous fish (Egerton et al. 2018; Kim et al. 2021), Proteobacteria, now Pseudomonadota Corrig. Phyl. Nov (Oren and Garrity 2021) resulted in the dominant phylum, followed by Firmicutes, now Fusobacteriota, which constitutes the second most abundant phylum in swordfish. The abundance of the phylum Proteobacteria in the guts of carnivorous fish seems to have a role in the digestion and absorption of proteins (Ray et al. 2012). In literature, there is no gut microbiota data to compare our findings in swordfish, but in cut swordfish, the study of (Indio et al. 2024) also reported Proteobacteria and Firmicutes as prevalent phyla observed by shotgun metagenomic. Instead, unlike literature findings on gut microbiota of carnivorous fish reporting facultative anaerobes, comprising *Vibrio, Pseudomonas, Acinetobacter, Corynebacterium, Alteromonas, Flavobacterium*, and *Micrococcus* as the predominant intestinal microbes (Wang et al. 2018), in our study, *Photobacterium* dominated the gut microbiota across all the weight categories and FAO fishing areas. This finding is in agreement with the comparison on dominant gut bacteria in 16 carnivorous fish species, in which *Photobacterium* was the second most frequently reported genus (Egerton et al. 2018) or highly abundant in Mackerel Icefish (Song et al. 2023) and Atlantic cod (Le Doujet et al. 2019). *Photobacterium* are Gram‐negative, aerobic members of the Vibrionaceae family and have been identified in fish gut microbiota as mutualistic bacteria that might aid chitin digestion of foods (Ray et al. 2012; Egerton et al. 2018; Song et al. 2023) and produce chitinases (Egerton et al. 2018) and harmful enzymes such as neuraminidases (Sugita et al. 2000). In general, Photobacteria occur free‐living in seawater and sediments or in interaction with marine animals (Urbanczyk et al. 2011), but certain species could be considered pathogenic for a broad range of marine hosts as well as a considerable problem in the fish food industry, representing specific spoilage organisms in chilled fish and seafood products (Dalgaard et al. 1997). The spoilage processes by *Photobacterium* involve the production of quantities of trimethylamine and biogenic amines such as histamine (Bjornsdottir‐Butler et al. 2018; Comas‐Basté et al. 2019), given that several species (*P. aquimaris*, *P. angustum*, *P. kishitanii, P. damselae*, and *P. phosphoreum* ) are known as histamine producers. Considering swordfish in terms of microbiological safety, *Photobacterium* resulted in the most abundant genera among the microbial populations identified by shotgun metagenomic in the swordfish flesh, significantly higher in swordfish in comparison to Tuna and also Salmon (Indio et al. 2024). In addition, swordfish cuts appeared the richest in terms of histamine‐producing bacteria, even if histamine and other biogenic amines were not quantified (Indio et al. 2024). Even though the *Xiphidae* family is not included among the families associated with a high amount of histidine by Regulation (EC) 2073/20005 for the food safety criteria histamine, two incidents of histamine outbreaks are described due to the consumption of imported smoked swordfish and swordfish fillets (Boutin et al. 1998; Chang et al. 2008).

In the observed swordfish gut microbiota belonging to adults, namely medium and larger individuals, the abundance of the genera *Pseudoalteromonas, Cetobacterium, Psychrilyobacter*, and *Psychrobacter* increases with the weight of the fish, conversely from juveniles in which these genera are always < 1%. This means that the gut microbiota of juveniles (smaller fish) is less rich and diverse than adults (medium and larger fishes). Also, the FAO fishing areas seem to affect the gut microbiota composition: the relative abundance of *Pseudoalteromonas, Psychrilyobacter*, and *Psychrobacter* genera was higher in FAO 34 and 37.1.1 areas, whereas *Cetobacterium* was higher only in the FAO 27 area. Due to their unique biological characteristics, we expected the Mediterranean and Atlantic swordfish stocks to have a distinct gut microbiota, but our findings give more evidence that the fish gut microbiota is primarily determined by the fish environment, rather than by genetic factors, as underlined by Kim and colleagues (2021). In literature on fish gut microbiota, *Cetobacterium* is reported to be a major candidate in *Oncorhynchus mykiss* (Etyemez and Balcázar 2015) and in five out of the six healthy edible fish species from intensive freshwater aquaculture, and to play a beneficial role in biochemical processes in the fish gut (Ofek et al. 2021). In addition, it is the dominant commensal bacterium in many fish species, and their metabolites are rich in vitamin B12, which is inferred to promote the health of fish (Xie et al. 2021). Also, *Psychrilyobacter* is reported as a key member genus with high relative abundance in the guts of marine invertebrates and animals, being present in over 20% of the intestines of European abalone (Liu et al. 2023). *Psychrobacter* can be recovered in high abundance from the guts of different marine fishes, namely four carnivorous coral reef fishes (Gao et al. 2020), *Salvelinus alpinus* (Ransom 2008), *Gadus morhua* (Dehler et al. 2017), and *Oncorhynchus mykiss* (Etyemez and Balcázar 2015). *Pseudoalteromonas, Cetobacterium, Psychrilyobacter*, and *Psychrobacter* are all Gram‐negative bacteria, of which *Pseudoalteromonas* and *Psychrobacter* are aerobic, whereas *Cetobacterium* and *Psychrilyobacter* are obligately anaerobic bacteria. They are all described in literature as seafood spoilage microbiota producing volatile organic compounds, causing musty off‐odours (Odeyemi et al. 2018).

In the end, the fish gut microbiota has become a frontier research field. Culture‐independent high‐throughput sequencing of 16S rRNA genes has greatly facilitated studies exploring microbial communities, such as the fish gut microbial compositions, generating large‐scale data sets that describe the microbial composition of a certain niche, data that could be used to understand and describe microbial diversity. But certain critical aspects need to be highlighted. Essential features of a bacterial community in a certain niche are characterised by the number of species present and their numerical composition, namely the bacterial diversity (Kim et al. 2017). This bacterial diversity is based on OTUs measurement, which can be defined at different levels of resolution, from phylum to genus and species, and can be quantified at two levels, namely the alpha (within‐sample) and beta (between‐sample) diversity. Alpha diversity metrics summarise the structure of a microbial community with respect to its richness (number of taxonomic groups in a sample), evenness (distribution of abundances of the groups), or both. Beta‐diversity metrics summarise which samples differ from one another by considering sequence abundances or considering only the presence or absence of sequences (Kers and Saccenti 2022). However, both diversity indices have specific biases, and therefore, it is essential to pinpoint that diversity is rather a simple statistic with which to describe the complexity of this microbial ecosystem. Studies employing a range of diversity measures reveal that the differences between them can be considerable, influencing the significance of results. This emphasises the need for a cautious approach when drawing conclusions from any one diversity index (Johnson and Burnet 2016). Due to the complexity of fish gut microbiota, the structure and function of the swordfish gut microbiota have not been investigated in depth. Collectively, these findings improve the understanding of this valuable fish and provide a reference for future studies of swordfish gut microbiota. Furthermore, considering that the gut is a primary source of bacterial contamination during animal slaughter and processing, leading to the potential presence of spoilage microorganisms and foodborne pathogens in food, a deeper characterisation of swordfish gut microbiota will also guide a better understanding of this valuable fish in terms of food safety.

## 5 Conclusions

This study provides the first characterisation of the gut microbiota of *Xiphias gladius* using next‐generation sequencing. In summary, our results offer a comprehensive view of the swordfish gut microbiota, derived from a significant sample size of 100 individuals caught in different FAO fishing areas, which were objects of this study (FAO 27, 34, 37.1.1 areas) and belonging to various size classes, enabling a detailed investigation of intra‐species diversity. Similar to other marine carnivorous fish species, the Pseudomonadota phylum dominated the gut microbiota, followed far behind by Fusobacteriota; however, differing from the literature, *Photobacterium* was found to be the most abundant genus. Differences in the alpha diversity were observed primarily between different weight categories rather than FAO fishing areas. Specifically, within‐species variability was more pronounced across size categories: smaller fishes exhibited a less rich and diverse gut microbiota with an almost exclusive presence of *Photobacterium* (98.5%) compared to medium and larger fishes, in which the genera *Pseudoalteromonas*, *Psychrilyobacter*, *Cetobacterium*, and *Psychrobacter* appeared to increase with fish weight. Although *Photobacterium* was dominant across all FAO fishing areas, distinctive microbial community compositions were found, namely the *Cetobacterium* in FAO 27 and *Pseudoalteromonas* in the other areas.

Since swordfish capture quota from both the Atlantic Ocean and the Mediterranean Sea is less than 30% with respect to the total (European Commission Mipaaft Feamp Unioncamere 2020), it would be interesting to study the gut microbiota of swordfish deriving from other FAO fishing areas, such as the Pacific and Indian Oceans, which together contribute to more than the 70% of the global capture. Moreover, in these areas, fishing techniques, the timing of stocking fish inside the boats before landing, production, conservation, and packing techniques in the factories are very different from the studied areas, and it could be interesting to verify if they influence the gut microbiota and, in case, the contamination of fish meat and of production environments.

The findings of this study enhance our understanding of the swordfish, a valuable fish much appreciated by EU consumers but also more vulnerable than other fish with regard to food safety. Further research is necessary to clarify the gut microbiota further and to understand the complete picture of the gut and flesh microbiota in swordfish.

## Author Contributions

**Alessandro Truant:** writing – original draft, visualization, investigation. **Federica Giacometti:** supervision, conceptualization, validation, writing – original draft. **Carmen Losasso:** formal analysis, visualization, writing – original draft, methodology, data curation, software. **Arianna Peruzzo:** formal analysis, visualization. **Sara Petrin:** formal analysis, visualization, data curation. **Irene Zancato:** visualization. **Vincenzo Di Leva:** visualization. **Valerio Giaccone:** visualization.

## Conflicts of Interest

The authors declare no conflicts of interest.

## Data Availability

The data that support the findings of this study are available on request from the corresponding author. The data are not publicly available due to privacy or ethical restrictions.
